# Genome Wide Analysis of Nucleotide-Binding Site Disease Resistance Genes in *Brachypodium distachyon*

**DOI:** 10.1155/2012/418208

**Published:** 2012-05-28

**Authors:** Shenglong Tan, Song Wu

**Affiliations:** ^1^Services Computing Technology and System Laboratory, Cluster and Grid Computing Laboratory, School of Computer Science and Technology, Huazhong University of Science & Technology (HUST), Luoyu Road 1037, Wuhan 430074, China; ^2^School of Information Management, Hubei University of Economics, Wuhan 430205, China

## Abstract

Nucleotide-binding site (NBS) disease resistance genes play an important role in defending plants from a variety of pathogens and insect pests. Many R-genes have been identified in various plant species. However, little is known about the NBS-encoding genes in *Brachypodium distachyon*. In this study, using computational analysis of the *B. distachyon* genome, we identified 126 regular NBS-encoding genes and characterized them on the bases of structural diversity, conserved protein motifs, chromosomal locations, gene duplications, promoter region, and phylogenetic relationships. EST hits and full-length cDNA sequences (from *Brachypodium* database) of 126 R-like candidates supported their existence. Based on the occurrence of conserved protein motifs such as coiled-coil (CC), NBS, leucine-rich repeat (LRR), these regular NBS-LRR genes were classified into four subgroups: CC-NBS-LRR, NBS-LRR, CC-NBS, and X-NBS. Further expression analysis of the regular NBS-encoding genes in *Brachypodium* database revealed that these genes are expressed in a wide range of libraries, including those constructed from various developmental stages, tissue types, and drought challenged or nonchallenged tissue.

## 1. Introduction

 To ward off the attacks of bacteria, fungi, oomycetes, viruses, and nematodes, plants have evolved various defense mechanisms to protect themselves. One of the major mechanisms is characterized by a gene-for-gene interaction that required a specific plant resistance (R) gene and a cognate pathogen avirulence (Avr) gene [1]. This type of specific resistance is often associated with a localized hypersensitive response, a form of programmed cell death, in the plant cells proximal to the site of infection triggered by the recognition of a pathogen product [2, 3]. Previous works show that the plant genomes contain a large number of R-genes to counter a variety of pathogens.

Most characterized R-genes contain the regions that encode NBS at the N-proximal part and a series of leucine-rich repeats (LRRs) at the C-proximal part [4]. The NBS domain is involved in signaling and includes several highly conserved and strictly ordered motifs such as P-loop, kinase-2, and GLPL motifs [5], which has been demonstrated by the binding and hydrolysis of ATP and GTP. However, the LRR motif is typically involved in protein-protein interactions and ligand binding with pathogen-derived molecules, suggesting that this domain may play a pivotal role in defining pathogen recognition specificity [6]. In plants, the NBS-LRR genes have been subdivided into two main groups based on the presence or absence of the N-terminal Toll/interleukin-1 receptor (TIR) homology region [7–9]. Most of those genes, especially in the monocots which lack the TIR, have a coiled-coil (CC) motif in the N-terminal region.

Previous studies show that the NBS-LRR class of genes is abundant in the plant species. So far, a large number of NBS-encoding sequences have been isolated from various plant species: 149 such sequences are present in the *Arabidopsis thaliana* genome [10], 535 in rice [11], 330 in poplar [12, 13], 333 in *Medicago truncatula* [14], 459 in *grapevine* [13], 55 in *papaya* [15], and 158 in *Lotus japonicus* [16, 17]. However, except a study which described the number of R-like genes and their evolutionary pattern among four different gramineous plants [18], no other information was reported about the NBS-encoding genes in the *Brachypodium distachyon *such as structural diversity and gene duplications. 

*Brachypodium* is a very attractive model system for the monocot lineage due to a number of favorable features, including its small stature, simple growth conditions, rapid life cycle, and genetic tractability [19, 20]. *Brachypodium* is a member of the subfamily Pooideae and is closely related to wheat, oats, and barley [21]. In addition to its obvious utility as a model for the world's most important food crops, *Brachypodium* is also a highly tractable model for emerging biofuel crops, such as switchgrass and Miscanthus [22]. In 2010, a draft sequence of the complete *Brachypodium* genome sequence (diploid-inbred line Bd21) was released [23]. This information is publicly accessible (http://www.brachypodium.org/) and is particularly useful for exploring gene families and predicting functional conservation between species.

In the present study, we performed a genome-wide analysis for the NBS-LRR resistance genes in *B. distachyon*. We identified a total of 239 NBS-encoding genes including 126 regular NBS genes and 113 nonregular NBS genes. Structural diversity, conserved protein motifs, gene duplications, chromosomal locations, phylogenetic relationships and promoter regions were analyzed in all the regular NBS-LRR-encoding genes to support their association. Meanwhile, expression analysis of the regular NBS-LRR genes in drought stresses and the tissue-specific libraries were carried out using the *Brachypodium* database. These results would facilitate the isolation of new resistance genes and offer more target genes to engineer more disease resistant crops.

## 2. Materials and Methods

### 2.1. Identification of NBS-LRR Genes

 The *B. distachyon* protein sequences (1.2 version) were downloaded from the website http://www.brachypodium.org/ to construct a local protein database. Method used to identify the NBS-encoding genes in *B. distachyon* is similar to that described in *Arabidopsis *and rice [10, 11]. The complete set of sequences from the NBS genes was identified in the genome of *B. distachyon* using a reiterative process. First, a set of candidate NBS genes with the NBS motif was selected from the complete set of predicted *B. distachyon* proteins using a hidden Markov model (HMM) [24] for the NBS domain from the Pfam database (PF00931; http://pfam.sanger.ac.uk/search). In the second step, the selected protein sequences were aligned based only on the NBS domain using CLUSTAL W [25]. This alignment was then used to develop a *B. distachyon*-specific HMM model to identify the *B. distachyon* R-like sequences according to the method used in *Arabidopsis* [10]. This step was crucial to find the maximum number of candidate genes. The refined HMM was then compared again with the complete set of predicted *B. distachyon* proteins. The threshold expectation value was set to 10^−10^ [11], a value determined empirically to filter out most of the spurious hits. And then, some gene models were manually modified if they lacked one or more of the conserved motifs characteristic of that class of NBS-LRR gene through gene prediction programs. In the third step, sequences of the predicted and manually modified NBS-containing proteins were compared to the nr (nonredundant) database by the BLASTP searches of the local database, allowing the identification of regular and nonregular NBS-genes [26]. Subsequently, the Pfam (http://pfam.janelia.org/), InterProScan (http://www.ebi.ac.uk/Tools/pfa/iprscan/), and PRODOM database (http://prodom.prabi.fr/prodom/current/html/home.php) were used to determine whether the corresponding NBS candidate proteins encoded the TIR, NBS, or LRR motifs by default. The COILS programs (http://www.ch.embnet.org/software/COILS_form.html and http://toolkit.tuebingen.mpg.de/pcoils) were used to specifically detect CC domains [27]. The detailed protein motif and domain information was used to classify the NBS-encoding genes into subgroups.

### 2.2. Analysis of the Conserved Motif Structures and Gene Duplication

 The structural diversity among the identified NBS genes was also investigated by us. The predicted aminoacid sequences were subjected to the domain and motif analyses. For this purpose, the NBS domain was defined as the region extending from the Pre-P-loop to the MHDV motif; which contains about 300 aminoacids, based on the Pfam. We performed the MEME (multiple expectation maximization for motif elicitation) analysis [28] on the 126 regular NBS-LRR genes from our predicted candidate proteins (Table 1) with the conditions: (1) optimum motif width was set to 6 and 50; (2) maximum number of motifs was designed to identify 20 motifs; (3) the iterative cycles were set by default. The nonregular genes were excluded from the MEME analysis because their sequences in NBS were too divergent or their motif lengths were too short to allow them to be aligned well with the regular NBS genes. Moreover, *B. distachyon* NBS gene-duplication events of the NBS genes were also investigated. We defined the gene duplication in accordance with the criteria: (1) the alignment covered >70% of the longer gene; (2) the aligned region had an identity >70%; (3) only one duplication event was counted for tightly linked genes [29]. A block of duplications was defined if more than one gene was involved in the duplication.

### 2.3. Chromosomal Locations of the NBS-LRR Genes and Phylogenetic Analysis

The starting positions of all the NBS genes were confirmed by Blat (http://genome.ucsc.edu/FAQ/FAQblat.html) search using a local database containing the complete *B. distachyon* genome sequences of each chromosome. The recursive algorithm and AWT package were subsequently used for the graphic portrayal of *B. distachyon* NBS-LRR genes. For the phylogenetic analysis, multiple alignments of the aminoacid sequences were performed by Clustal W with default options and then by Gblocks [30] for manual corrections of the alignments. The phylogenetic trees were constructed based on the bootstrap neighbor-joining (NJ) method with a Kimura two-parameter model by MEGA [31]. 

### 2.4. Identification and Analysis of the Promoter Regions

For each predicted regular NBS gene, the 2 kb upstream regions were selected according to the position of the genes provided by the *B. distachyon* annotation information. The extracted sequences were screened against the PLACE database [32]. Regulatory elements overrepresented in the dataset and known to be involved in regulation during the resistance response and under stressed conditions were selected for further analysis [33]. Among them, WBOX (sequence TGAC(C/T)) associated with the WRKY transcription factors [34], CBF (GTCGAC) [35], and GCC boxes associated with the ERF-type transcription factors [36] were retained for further analysis.

### 2.5. Expression Analysis for the Regular NBS-LRR Candidate Genes

To gain the insight into the expression profiles of NBS-LRR genes in *B. distachyon* in different tissues and tissues under the drought stress levels, the *B. distachyon* EST database was searched using the identified NBS-LRR genes. The data thus obtained was analyzed and grouped according to the level of stress exposure and tissue specificity.

## 3. Results

### 3.1. Identification and Classification of the NBS-Encoding Genes

**Figure 1:**
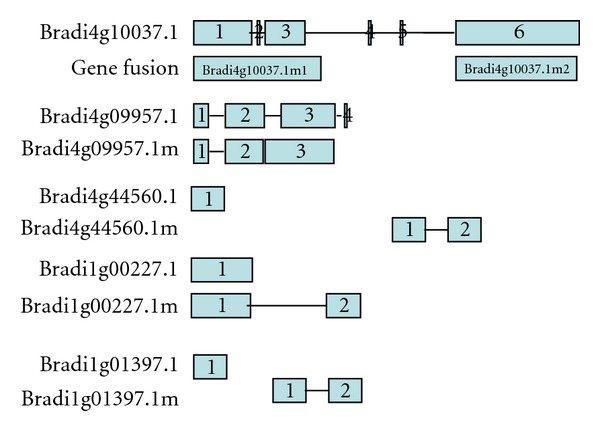
Manual modification of six gene models. Exons are drawn approximately to scale as shading boxes; connecting thin lines indicate the positions of introns, which are also drawn to scale.

 Availability of the complete* B. distachyon* genome sequences has made it possible for the first time to identify all the NBS-encoding genes in this plant species. From the first two steps of filters, a total of 239 NBS-encoding genes were identified in the *B. distachyon *(Supplemental File 1 and Supplemental File 2). 6 gene models were improved through manual modification, which were indicated with an “m” beside the gene name (Figure 1). For each revised gene model, the number of introns, exons and their positions on the genome were determined by BLASTN search using a local database containing the complete *B. distachyon* genome sequences of each chromosome (Supplemental File 3). Among of the six gene models, the sequence of Bradi4g09957.1 m matched perfectly with the accession number ACF22730.1 of GenBank and was thought to have a wrong terminal exon. Bradi4g44560.1, Bradi1g00227.1, and Bradi1g01397.1 were predicted as those which lacked specific motifs or contained large deletions compared with conventional NBS-RR genes even though they had apparently intact ORFs. For example, Bradi1g00227.1 lacked a C-terminal of the predicted protein as a result of a deletion at the 3′end of the gene. Bradi4g10037.1 was thought as the gene fusion of Bradi4g10037.1m1 and Bradi4g10037.1 m2. Through searching the *B. distachyon* EST database, we found that all the revised gene models were supported by EST evidence.

 In the third step, we determine whether the identified R-like genes belonged to regular or nonregular genes in accordance with the criteria used in rice [11]: (1) The alignment covered ≥70% of the longer gene; (2) The aligned region had an identity ≥50%. Through the comparison of nr database we considered 126 hits as the regular NBS genes which primarily showed ≥50% identity with the subject sequence of nr database, and the remaining hits were defined as the nonregular NBS-encoding genes. Although the nonregular genes contained the NBS structure, they were notably different from the regular NBS genes because of excessively short motif lengths or too divergent motifs. Thus, we restricted our current analysis to the 126 regular NBS-LRR genes

NBS domains of the NBS genes in *A. thaliana* could be phylogenetically classified into two distinct groups, distinguished by the presence and absence of a TIR motif in the N-terminal regions [9]. However, in the *B. distachyon* genome, none of them contained the TIR domain. Therefore, the sequences within NBS domains in regular NBS genes could not give a clear classification for these genes. For these reasons, we classified the regular NBS genes in *B. distachyon* based on the N-terminal and LRR regions. As the non-TIR genes from dicots typically have a CC motif in the N-terminal region, we first identified 113 of 126 regular NBS-LRR genes with CC motif from *B. distachyon*. In addition, some of the regular NBS-LRR genes contained some unknown motifs, which were symbolized as X. According to the differences of the regular NBS-LRR genes in the N-terminal and LRR regions, we finally classified them into four types: CNL, NL, CN, and XN (Table 1).

### 3.2. Analysis for the Conserved Motif Structures in the Regular NBS-LRR Genes

**Figure 2:**
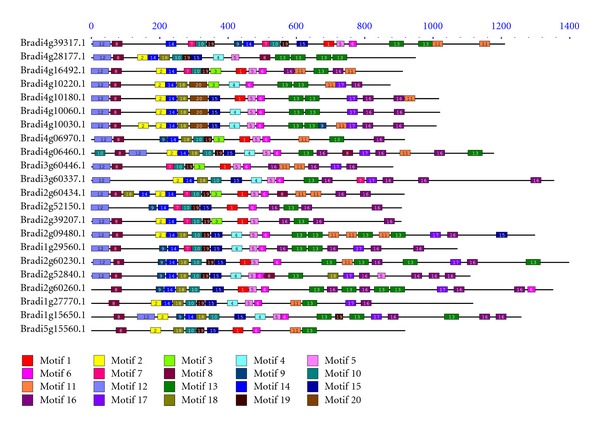
Examples of summarized and aligned MEME motifs for different domains of CNL proteins. All proteins were displayed in the Supplementary Material (Supplementary File 4 available online at doi:10.1155/2012/418208).

To investigate whether the N-terminal region in regular NBS genes shared motifs, and to examine whether the CC and non-CC gene groups also shared motifs the genes and gene groups were analyzed together using the program MEME. And, as expected, 20 putative conserved motifs were found (Figure 2; Supplemental File 4) among them. The detailed motif sequences were shown in Table 2.

The MEME results revealed that the N-terminal of NBS-LRR genes was not very divergent as compared with those of rice [11]. All the CNL and CN genes at the N-terminal contained a Q(L/I/V)RD motif (Table 2; motif 8), as a non-TIR motif that was present in nearly all the NBS-LRR genes of different plant species [11]. And only one gene (Bradi2g09434.1) among the remaining genes of other types contained this conserved motif in the middle of its sequence.

Previous work identified eight major motifs in the NBS region, and most of them have different patterns depending on whether they are present in the TNL or CNL groups [7]. In this study, the MEME results identified the motifs that matched the eight major motifs identified previously confirming that the NBS domain is the most conserved region among the domains encoded by R genes. Intriguingly, the eight motifs identified in *B. distachyon* were in the same order with that found in the *A. thaliana*. And the P-loop, Kinase-2, RNBS-B,GLPL and MHDV motifs showed high levels of similarity between the R-like genes in *B. distachyon* and* A. thaliana*, whereas RNBS-A, RNBS-C, RNBS-D, and RNBS-E in the *B. distachyon* were quite dissimilar to their counterparts in *A. thaliana* (Table 2). However, the eight major motifs differed in their divergence within and between the 102 CNL, 12 NL, 11 CN, and 1 XN groups. In addition, a previous study showed that GLPL motif was the core conserved domain of R genes [7]. In this study, we found that 96 percent of the regular NBS-LRR genes contained this motif in *B. distachyon*.

### 3.3. Duplications of NBS Genes

**Figure 3:**
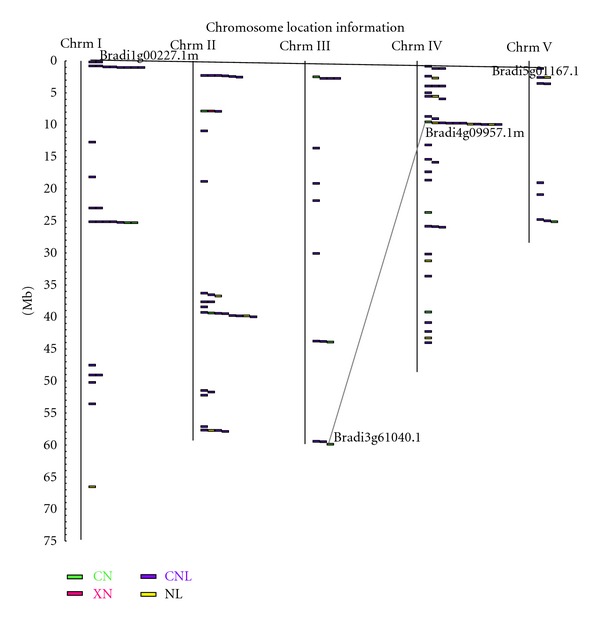
Distribution of the regular NBS encoding genes on the *B. distachyon *chromosomes. The scale is in megabases (Mb). Grey straight line connects the NBS genes present on duplicate chromosomal segments.

 During evolution, both segmental duplication and tandem duplication have contributed to the large number of gene families in plants [37]. The gene duplications have greatly expanded the NBS gene family in both monocot and eudicot lineages. In this study, we confirmed these genome duplications by the BLAST comparison of all the predicted *B. distachyon* proteins against each other. A total of 49 out of the 126 regular NBS genes duplications were identified and were subsequently divided into 20 gene families. The maximum number of family members was seven, and the average number of family members was 2.45. More robust analysis of gene duplication in NBS-LRR genes was carried out by comparing recent duplications of the NBS-encoding genes in *Arabidopsis*, rice, and *B. distachyon*. Previous study showed that 472 regular R-like genes were found in rice [11]. When more stringent criteria were applied, 464 regular R-like genes in rice were identified by Yang et al. [38]. Here, the analysis of gene duplication among the three plant genomes was restricted to their regular R-like genes. The results revealed that the percentage of the multigene families (two or more members per family) in regular NBS genes of *B. distachyon* (38.9%) genome was significantly lower than in *Arabidopsis* and rice (46.6 and 53.4%, resp.) genomes (Table 3). Furthermore, the number of family members in *B. distachyon* (20) was lower than both of the *Arabidopsis* and rice (25 and 93, resp.). The average number of NBS members per multigene family was 2.45 in *B. distachyon*, and also lower than that of *Arabidopsis* (3.24) and rice (3). This analysis revealed reduced duplications in the genome and multigene families in *B. distachyon.* More interestingly, only two pair of NBS-LRR genes (Figure 3) was found on duplicated chromosomal segments. Thus, tandem duplication could play a major role in the expansion of NBS-encoding genes in *B. distachyon.*

### 3.4. Chromosomal Locations and Phylogenetic Analysis of the Regular NBS-LRR Genes

Figure 3 shows the locations of the regular NBS-LRR genes on the 5 chromosomes of *B. distachyon*. They were separately located on each chromosome individually or in clusters, and their distribution was non-random (Figure 3; Supplemental File 5). For example, Chromosome 5 contains only 10 NBS-LRR genes, while Chromosome 4 contains about one-third of the total regular NBS-LRR genes. There was no obvious difference between the distributions of the CC-and non-CC-types of genes on the chromosomes. Studies on *Arabidopsis* and rice report uneven chromosomal distribution of the NBS-encoding genes, and most of the NBS-containing genes have been found in clusters [9, 11]. Based on Houb's (2001) [39] definition of a gene cluster, it is a region that contains four or more genes within 200 kb or less. In this study, we found 43 genes (51%) resided in 11 gene clusters using a sliding window size of 200 kb, and that the average number of genes in a cluster was 4 (Supplemental File 5). For this window size, two largest clusters contained 7 genes. One was on chromosome 1, and the other was on chromosome 4. However, no gene cluster was found on chromosome 3 and 5. If a sliding window size of 100 kb was used, 69% of NBS domains occurred in clusters of at least two genes, which was much lower than that in *M. truncatula* (79.8%) [14]. Further relaxing these clustering criteria with sliding window size of 430 kb, a significant fraction of *B. distachyon* NBS were in two very large, extended clusters: one was at end of chromosome 1 containing eight genes, and another was at the end of chromosome 4 containing 11 genes.

### 3.5. Phylogenetic Analysis of the Regular NBS-LRR Encoding Genes

**Figure 4:**
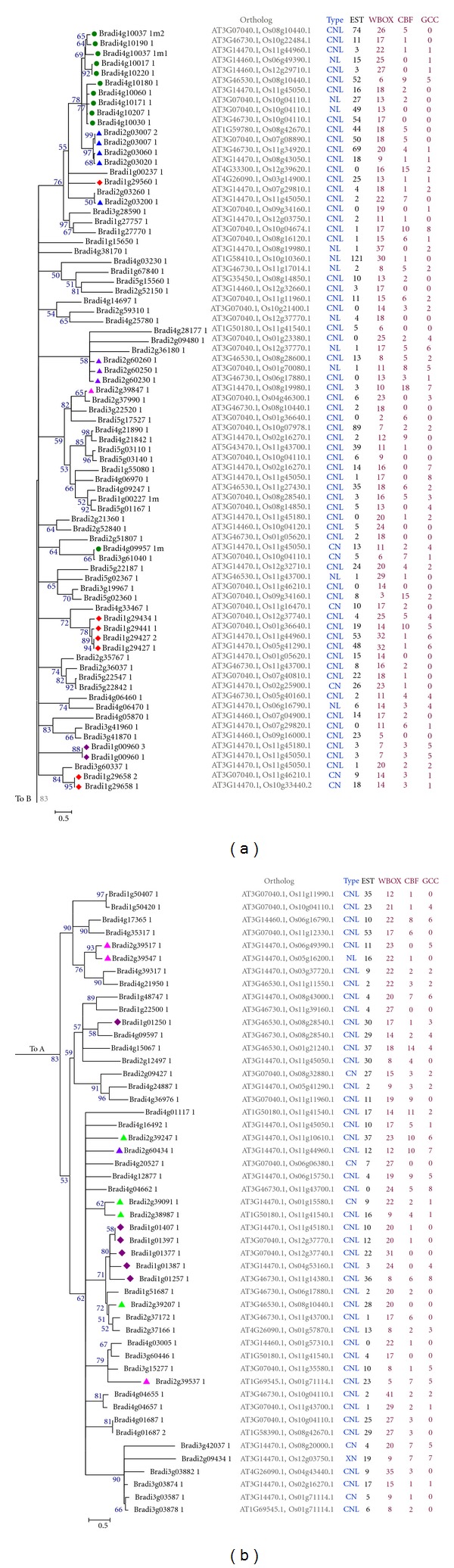
Phylogenetic tree derived from 126 regular NBS encoding genes in* B. distachyon*. Chromosomal origin of each gene is indicated in the sixth character (Bradi4, etc.) of each sequence name. Bootstrap values for important basal clades are indicated in black beside the branch. Different color patternings indicate different gene clusters or superclusters. The genes belonging to duplicated chromosomal segments were indicated with their gene names in blue. Columns on the right side show orthologs, domain configurations, numbers of EST, and predicted regulatory elements (Supplemental File 1).

 The phylogenetic relationships, among the regular NBS genes and the evolutionary history of this gene family were inferred by constructing a combined phylogenetic tree with the aligned regular R-like protein sequences. For the size of the inferred tree image was too large, we divided it into two parts (Figures 4(a) and 4(b)) on the basis of the clades constructed from* B. distachyon* R-like genes.  Figure 4 showed phylogenies, including chromosome of origin (by sequence name), gene relatedness, gene ortholog in *A. thaliana* and rice, evolutionary rate, approximate expression levels, and the regulatory element counts (Right). The phylogenetic tree also showed the gene clusters of *B. distachyon* with labels before the gene name in different color patterning. For example, the majority of members in the largest gene supercluster on chromosome 4 (by green circle patterning) happened to cluster together in the phylogenetic tree. However, the rest of gene clusters was not the case.  Figure 4 also revealed the genes due to chromosomal duplicate with their name in blue. For example, Bradi4g09957.1 m and Bradi3g61040.1 are from different chromosome, and they had 88.48% identification between their aminoacid sequences, which showed that these genes were possibly originated from genomic duplication and subsequent divergence under the selective pressure of pathogens. In addition, we found that 111 of these 126 R-like genes from different chromosomes are the orthologs of *A. thaliana *from the same chromosome 3, which showed that the 111 R-like genes from *B. distachyon* and the chromosome 3 of *A. thaliana* may have evolved from a common ancestral gene via speciation. However, it was not the case in rice. Even the members from the same gene family of *B. distachyon* had high similarity in protein sequences, their orthologs in rice were still from different chromosomes. That may be the reason that *A. thaliana *and Poaceae split far before the separation of *B. distachyon *and rice.

### 3.6. *In Silico* Analysis of the Promoter Regions of the NBS-Encoding Genes

 We identified the promoter sequences in 2 kb windows upstream of the predicted regular NBS-encoding genes as described in *M. truncatula *[14]. Three regulatory elements including the WBOX cassettes, CBF boxes, and the GCC motif implicated in either response to pathogens or plant stress were identified as being overrepresented in the 2 kb region upstream of regular NBS-LRRs. The analysis results showed that WBOX element existed in all the regular NBS encoding genes averaging 17.10 per gene (Supplemental File 1; Figures 4(a) and 4(b)) and 80.95% contained at least 11 predicted WBOXs. In contrast, the average numbers of other element types were 3.36 (CBF) and 2.01 (GCC). Of the predicted regular NBS-LRR genes, 51.6% contained the three multiple boxes. However, we see no clear evidence of a correlation between the arrangement of these promoter cassettes (WBOX, CBF, and GCC) and *in silico* expression via EST counts.

### 3.7. *In Silico* Analysis of the Expression of NBS Encoding Genes Based on the *B. distachyon* EST Database

To assess which genes in this study had expression support, the coding regions of the regular NBS-LRR genes were searched against EST databases of *B. distachyon* with BLASTN. The matches with 90% identity were considered to be significant. Our analysis revealed that only 8 percent of the 126 regular NBS-LRR genes were not supported by EST from *B. distachyon* tissue-specific and drought stress-related libraries (Supplemental File 1). This result indicated that either the 10 NBS-LRR genes were not expressed in all the conditions used for analysis or that they were expressed in very low quantity, which cannot be detected. Meanwhile, the large proportion of genes with expression support testified to the high quality of the initial genome annotation and provided very useful information for further experimental verification.

## 4. Discussions

 Many aspects of the NBS disease resistance gene family have been extensively studied and described in other species [10–17]. In this study, we identified 239 NBS-encoding genes in the 1.2 version of the *B. distachyon* Bd21 genome, which represents 0.77% of all the predicted proteins. The number of NBS-encoding genes was consistent with that of *Arabidopsis* Col-0 genome [10], but clearly lower than that of the rice genome [11]. More interestingly, the same number of NBS-encoding genes was identified by Li et al. [18] from the 1.0 version of the Bd21 genome when they analyzed the unique evolutionary pattern of numbers of gramineous NBS-LRR genes. However, the method used by them for estimating the number of NBS-LRR genes does not allow us to repeat, thus we could not investigate the relationship between these data. But at least there was some difference between them just judged from the descriptions of the two papers. For example, in our study six gene models were manually modified by us, but they did not mention that in their articlepaper. On the other hand, the types classified by the domain of these R-like genes and the number of each type were different, which may be caused by the parameters set of our analytic software. In sum, there was little in common between our studies except for estimating the same number of R-like genes in* B. distachyon*. They focused on research on the evolutionary relationship of the R-like genes among the four grass species, while our focus is to characterize these identified R-like genes of* B. distachyon* in detail on the bases of structural diversity, conserved protein motifs, chromosomal locations, gene duplications, *in silico* gene expression, promoter region, and phylogenetic relationships.

 In addition, in order to examine whether our method is feasible in identification of NBS-like genes from *B. distachyon* Bd21, we manually counted all of the annotated NBS-like proteins from NCBI. Only two NBS genes (accession numbers GU733187 and ACF22730.1) in GenBank were found on Chromosomes 2 and 4 separately, which corresponded with the Bradi2g51807.1 and Bradi4g09957.1 m identified by us. More interestingly, one of these two genes was misannotated during the automated annotation process. Therefore, we undertook the complete manual reannotation and analysis of the NBS-LRR gene family to rectify incorrect start codon predictions, splicing errors, missed or extra exons, fused genes, split genes, and incorrectly predicted pseudogenes. At last six gene models were modified for which the translation of predicted protein sequences did not match other annotated conventional NBS-LRR proteins. For example, the sequence of Bradi4g09957.1 m mentioned above was reannotated with the wrong terminal exon, which matched perfectly with the accession number ACF22730.1 of GenBank. And the sequences of Bradi4g10037.1m2, Bradi1g00227.1 m and Bradi4g44560.1 m were reannotated with either incorrect start codon predictions or deleted protein motifs or domains, which were identical to the *B. distachyon* sequences of XP_003577237.1, XP_003558418.1, and XP_003577143.1 predicted by NCBI (released on 15 November, 2011), separately. However, these three sequences were not annotated by the http://www.brachypodium.org/. *In silico* gene expression showed that they all have EST support, especially the gene model Bradi4g10037.1m2 which was supported by 74 ESTs. Similarly, One-third of the identified R-like genes were found in *Arabidopsis* [10], and 59 identified R-like genes were found in *M. truncatula* [14] with annotation errors. Therefore, analyses using only automated annotations without manual reassessment risk misinterpretation, particularly when large gene families are considered. Continual refinements to gene prediction programs may reduce the rate of errors in annotation.

### 4.1. Domain Structures

 The three-dimensional structures of plant resistance proteins were based on research on their animal homologs, but advanced technologies in molecular biology and bioinformatics tools have enabled prediction of the structures and mechanisms of interaction of specific receptors with pathogen effectors [40]. The two main domains of plant R proteins, NBS and LRR, seem to be the most crucial in the pathogen recognition process and the activation of signal transduction in the response to pathogen attack. The NBS domain was characterized by NTPase activity, and it was suggested to play a crucial role as a molecular switch activating signal transduction. Several conservative motives such as the P loop (Walker A or kinase 1), the RNBS-A, kinase 2 (Walker B), RNBS-B, RNBS-C, GLPL, RNBS-D, and MHD motifs can be distinguished in this domain [41]. Recently, NBS motif has been used as a molecular marker to assess variation associated with potentially functional regions of the genome underlying specific phenotypes [42]. And the essential structural element of the LRR domain is the tandem repeat of 20–30 aminoacids containing a consensus sequence LxxLxLxxNxL, where L is a leucine residue or another aliphatic aminoacid, N is asparagin, threonin, serine or cystein, and x is any aminoacid [43–45]. A protein with an LRR domain has to contain at least two LRR repeats. The tertiary structure of a single LRR domain is usually a horseshoe-shaped superhelix, and each repeat forms other coils of the superhelix. It is believed that LRR domains constitute a platform for protein-protein interactions [44, 46].

 Multiple alignment and MEME analysis among the 126 regular R-like genes in *B. distachyon* revealed that the presence of conserved domains such as P-loop (motifs 2 and 9), RNBS-A (motifs 14), Kinase2 (motifs 7 and 18), RNBS-B (motif 10), GLPL (motifs 3 and 15), RNBS-C (motif 19), RNBS-D (motif 1), and MHDV (motif 6) (Table 2). From the MEME results, we found that not all the regular genes contained these eight conserved motifs but at least five of them. As for LRR domain, no significant motif was found like the motifs that were in the NBS domain from our meme results because the rates of aminoacid substitutions in LRR domains were generally high. Paterson et al. was the primary contributor to the evolution of resistance genes, in that the beta sheets in this core region have more potential for functional innovation than those in other regions [47]. For CC-motif, the analytic result of the Coils program was consistent with that of MEME. All the regular R-like genes with CC-motif at the N-terminal of sequences included the motif 8 (Table 2). The domain analysis of these regular R-like genes also showed that a majority of these genes contained the three conserved domains mentioned above: CC, NBS, and LRR, which belonged to the canonical classes described in the literature [9, 10]. And a minority of genes had less typical or atypical domain arrangements. For example, Bradi2g09434.1 lacked the LRR domain but contained a C-terminal AP2 domain. Bradi5g22187.1 (CNL) contained a zef-BED domain at the C-terminal of its sequence like in *Populus* [12], and there were also several genes with atypical structure CNNL. They were uniformly distributed on different chromosomes, supported by ESTs but were not included in any multigene families or gene clusters.

### 4.2. Gene Distribution, Gene Duplication, and Phylogenic Analysis

 As is the case in other plant genomes, NBS genes are also clustered physically in *B. distachyon*. 11 gene clusters with different members are nonuniformly distributed on chromosomes 1, 2, and 4 of *B. distachyon*. This is clearly an outcome of the birth and death process that results from tandem duplication or contraction in a cluster. Not only the NBS-LRR genes of* B. distachyon* tended to cluster, but many also lay in superclusters, such as a supercluster with 11 NBS genes on the terminal of chromosome 4. It was also found in *M. truncatula,* whose largest supercluster included 82 genes. These examples demonstrated the parallel expansion of both gene copy number and diversity between the copies in a gene cluster. Of course there are also some of NBS-LRR genes which did not cluster together with any other NBS genes in *B. distachyon. *These genes are singletons, some of which are closely related to sequences elsewhere in the genome, such as Bradi4g03005.1 and Bradi3g60446.1. Although they are rare, these genes may play the role of pioneers, seeding new regions of the genome with NBS-LRRs, and potentially establishing new locations for future clusters.

 Phylogenetic analysis of the 126 regular R-like genes showed that all the members of each multigene family identified by us clustered together in the phylogenetic tree. But it was not the case in their members of our identified gene clusters. We found that most clades of the phylogenetic tree were dominated by sequences from one chromosome (and usually from one or a small number of genomic clusters), but many also contained small numbers of sequences from other chromosomes. These mixed clades could arise in several ways: by chromosomal rearrangement (e.g., breakage and fusion), by transposition, or by large-scale genomic duplication. Examples were shown in Figure 4, such as the mix clades which contained a subclade with ten sequences from chromosome 4 and a subclade with 4 sequences from chromosome 2 (at the top of Figure 4(a)). The presence of heterogeneous phylogenetic NBS clusters in *B. distachyon* resembles the situation in rice [48] and *Arabidopsis* [10].

### 4.3. Promoter Region and EST Expression

 To examine the expression levels of the regular NBS-LRR candidates from different tissues of *B. distachyon*, each candidate was analyzed using *B. distachyon* EST database. These genes are expressed in a wide range of libraries, including those constructed from various developmental stages, tissue types, and drought challenged or nonchallenged tissues. Only 8% of the regular NBS genes had no EST support, which reflects probably the low levels of expression or the fact that the NBS genes are only expressed under specific conditions in specific tissues. Thus, it should be confirmed by further experimental studies.

Besides this, the 2,000 bp upstreams of the NBS-LRR genes were also examined by us. WBOX motifs have been described upstream in the NPR1 gene (a positive regulator of inducible plant disease resistance [49] and upstream of most *Arabidopsis* pathogen response genes [50]). The results showed each regular NBS-encoding gene contained this regulatory element averaging 17.10 per gene, which is much higher than that of *M. truncatula*. There was no significantly correlation between the number of EST and WBOX motifs in each gene. And the counts of EST and WBOX motifs vary substantially between clades and even between highly similar genes within the same clade of the phylogenetic tree.

## Supplementary Material

 Supplemental file 1. The summary information of 126 regular NBS-LRR genes. Supplemental file 2. The summary information of 113 non-regular NBS-LRR genes. Supplemental file 3. The six modified gene models. Supplemental file 4. The conserved motifs of 126 regular NBS-LRR genes. Supplemental file 5. The gene clusters of 126 regular NBS-LRR genes.

## Figures and Tables

**Table 1:** The number of genes that encode domains similar to NBS genes in two gramineae genomes.

Predicted protein domain	Letter code	*B. distachyon*	Rice^a^
Regular NBS-LRR type genes			
CC-NBS-LRR	CNL	102	160
NBS-LRR	NL	12	0
X-NBS-LRR	XNL	0	264
NBS-LRR from TMRI	CNL and XNL	0	16

Total		114	440

Regular NBS type genes			
CC-NBS	CN	11	7
X-NBS	XN	1	25

Total regular NBS genes		126	472

Nonregular NBS genes			
CC-NBS-LRR	CNL	55	0
NBS-LRR	NL	4	
X-NBS-LRR	XNL	2	40
CC-NBS	CN	37	0
X-NBS	XN	3	20
NBS	N	12	0
TIR-NBS	TN	0	3

Total nonregular NBS genes		113	63
Total NBS-LRR genes		175	480
Total NBS genes		239	535

Note: ^a^Data from Zhou et al. [11].

**Table 2:** The motif sequences identified by the MEME.

NO.	Best possible match	NBS motif
1	T**CLLYLSAFPED**YEIERERLVRRWIAEGF	RNBS-D
2	VRKLNVVSIVGF**GGLGKTTLA**KQVYDKIR	P-loop
3	CPDMFKEVSNE**ILKKCGGLPLAI**ISISSL	GLPL
4	**ALYLSYDELPHHLK**QCFLYCALYTEDSII	RNBS-C
5	EETAEEYYYELIHRNLLQPDG	—
6	AC**RVHDMVLDLICSLSSEE**NF	MHDV
7	FLKD**KRYLIVIDDIW**STSAWR	Kinase-2
8	NDTVRTWVKQVRDLANDVEDCLLDFVLYS	—
9	VLSIV**GFGGLGKTTLAK**AVYR	P-loop
10	IKCAFPDNEKGS**RIIITTR**NEDVANICCC	RNBS-B
11	NLRYIGLRRTNVKSLPDSIENLSNLQTLD	—
12	VSAADGALGPLLGKLATLLAEEYSRLKGVRGEIRSLKSELTSMHGALKKY	—
13	IQTIPDCIANLIHLRLLNLDGTEISCLPESIGSLINLQILN	—
14	GSFNIQAWVCVSQDYN**EVSLLKEVLR**NIG	RNBS-A
15	SAHPNLEIIGMEIVKKLK**GLPLA**AKAIGSLL	GLPL
16	PPLWQLPNLKYLRIEGAAAVTKIGPEFVG	—
17	QLRPPGNLENLWIHGFFGRRYPTWFGTTF	—
18	QGETIGELQRKLAETIEGKS**FFLVLDDVW**	Kinase-2
19	IYRMK**PLSDDY**SRRLFYKRIF	RNBS-C
20	LRTPLHATTAGVI**LVTTR**DDQIAMRIGVEDIHRVDLMSVEVGWELLWKSM	RNBS-B

Note: The bolded sequence indicates the conserved NBS domain sequences.

**Table 3:** Comparison of duplications in the NBS-encoding R genes from the three plant genomes.

Organization	*B.distachyon*	*Arabidopsis* ^ b^	Rice^a^
Single-genes	77	93	216
Multigenes	49	81	248
Number of family members	20	25	93
Maximal family members	7	7	10
Average members per family	2.45	3.24	2.67
Multigenes/single-gene families	0.64	0.87	1.14
Percentage of multigene families	38.9%	46.6%	53.4

Note: ^a^Data from Zhou et al. [11].

^b^Data from Meyers et al. [10].

## References

[1] Flor HH (1971). Current status of the gene-for-gene concept. *Annual Review of Phytopathology*.

[2] Dangl JL, Dietrich RA, Richberg MH (1996). Death don’t have no mercy: cell death programs in plant-microbe interactions. *Plant Cell*.

[3] Heath MC (2000). Hypersensitive response-related death. *Plant Molecular Biology*.

[4] Hulbert SH, Webb CA, Smith SM, Sun Q (2001). Resistance gene complexes: evolution and utilization. *Annual Review of Phytopathology*.

[5] Belkhadir Y, Nimchuk Z, Hubert DA, Mackey D, Dangl JL (2004). Arabidopsis RIN4 negatively regulates disease resistance mediated by RPS2 and RPM1 downstream or independent of the NDR1 signal modulator and is not required for the virulence functions of bacterial type III effectors AvrRpt2 or AvrRpm1. *Plant Cell*.

[6] Ellis JG, Lawrence GJ, Luck JE, Dodds PN (1999). Identification of regions in alleles of the flax rust resistance gene L that determine differences in gene-for-gene specificity. *Plant Cell*.

[7] Meyers BC, Dickerman AW, Michelmore RW, Sivaramakrishnan S, Sobral BW, Young ND (1999). Plant disease resistance genes encode members of an ancient and diverse protein family within the nucleotide-binding superfamily. *Plant Journal*.

[8] Pan Q, Wendel J, Fluhr R (2000). Divergent evolution of plant NBS-LRR resistance gene homologues in dicot and cereal genomes. *Journal of Molecular Evolution*.

[9] Richly E, Kurth J, Leister D (2002). Mode of amplification and reorganization of resistance genes during recent *Arabidopsis thaliana* evolution. *Molecular Biology and Evolution*.

[10] Meyers BC, Kozik A, Griego A, Kuang H, Michelmore RW (2003). Genome-wide analysis of NBS-LRR-encoding genes in *Arabidopsis*. *Plant Cell*.

[11] Zhou T, Wang Y, Chen JQ (2004). Genome-wide identification of NBS genes in japonica rice reveals significant expansion of divergent non-TIR NBS-LRR genes. *Molecular Genetics and Genomics*.

[12] Kohler A, Rinaldi C, Duplessis S (2008). Genome-wide identification of NBS resistance genes in *Populus trichocarpa*. *Plant Molecular Biology*.

[13] Yang SH, Zhang XH, Yue JX, Tian DC, Chen JQ (2008). Recent duplications dominate NBS-encoding gene expansion in two woody species. *Molecular Genetics and Genomics*.

[14] Ameline-Torregrosa C, Wang BB, O’Bleness MS (2008). Identification and characterization of nucleotide-binding site-leucine-rich repeat genes in the model plant Medicago truncatula. *Plant Physiology*.

[15] Porter BW, Paidi M, Ming R, Alam M, Nishijima WT, Zhu YJ (2009). Genome-wide analysis of *Carica papaya* reveals a small NBS resistance gene family. *Molecular Genetics and Genomics*.

[16] Li XY, Cheng HY, Ma W, Zhao Y, Jiang H, Zhang M (2010). Identification and characterization of NBS-encoding disease resistance genes in *Lotus japonicus*. *Plant Systematics and Evolution*.

[17] Tamura M, Tachida H (2011). Evolution of the number of LRRs in plant disease resistance genes. *Molecular Genetics and Genomics*.

[18] Li J, Ding J, Zhang W (2010). Unique evolutionary pattern of numbers of gramineous NBS-LRR genes. *Molecular Genetics and Genomics*.

[19] Draper J, Mur LA, Jenkins G (2001). Brachypodium distachyon. A new model system for functional genomics in grasses. *Plant Physiology*.

[20] Opanowicz M, Vain P, Draper J, Parker D, Doonan JH (2008). Brachypodium distachyon: making hay with a wild grass. *Trends in Plant Science*.

[21] Kellogg EA (2001). Evolutionary history of the grasses. *Plant Physiology*.

[22] Bevan MW, Garvin DF, Vogel JP (2010). Brachypodium distachyon genomics for sustainable food and fuel production. *Current Opinion in Biotechnology*.

[23] Vogel JP, Garvin DF, Mockler TC (2010). Genome sequencing and analysis of the model grass Brachypodium distachyon. *Nature*.

[24] Eddy SR (1998). Profile hidden Markov models. *Bioinformatics*.

[25] Thompson JD, Higgins DG, Gibson TJ (1994). CLUSTAL W: improving the sensitivity of progressive multiple sequence alignment through sequence weighting, position-specific gap penalties and weight matrix choice. *Nucleic Acids Research*.

[26] Altschul SF, Gish W, Miller W, Myers EW, Lipman DJ (1990). Basic local alignment search tool. *Journal of Molecular Biology*.

[27] Lupas A, Van Dyke M, Stock J (1991). Predicting coiled coils from protein sequences. *Science*.

[28] Bailey TL, Elkan C (1995). The value of prior knowledge in discovering motifs with MEME.. *Proceedings of the International Conference on Intelligent Systems for Molecular Biology*.

[29] Gu Z, Cavalcanti A, Chen FC, Bouman P, Li WH (2002). Extent of gene duplication in the genomes of Drosophila, nematode, and yeast. *Molecular Biology and Evolution*.

[30] Castresana J (2000). Selection of conserved blocks from multiple alignments for their use in phylogenetic analysis. *Molecular Biology and Evolution*.

[31] Tamura K, Dudley J, Nei M, Kumar S (2007). MEGA4: molecular evolutionary genetics analysis (MEGA) software version 4.0. *Molecular Biology and Evolution*.

[32] Higo K, Ugawa Y, Iwamoto M, Korenaga T (1999). Plant cis-acting regulatory DNA elements (PLACE) database: 1999. *Nucleic Acids Research*.

[33] Jang CS, Kamps TL, Skinner DN, Schulze SR, Vencill WK, Paterson AH (2006). Functional classification, genomic organization, putatively cis-acting regulatory elements, and relationship to quantitative trait loci, of sorghum genes with rhizome-enriched expression. *Plant Physiology*.

[34] Dong J, Chen C, Chen Z (2003). Expression profiles of the *Arabidopsis* WRKY gene superfamily during plant defense response. *Plant Molecular Biology*.

[35] Sakuma Y, Maruyama K, Qin F, Osakabe Y, Shinozaki K, Yamaguchi-Shinozaki K (2006). Dual function of an Arabidopsis transcription factor DREB2A in water-stress-responsive and heat-stress-responsive gene expression. *Proceedings of the National Academy of Sciences of the United States of America*.

[36] Ohme-Takagi M, Suzuki K, Shinshi H (2000). Regulation of ethylene-induced transcription of defense genes. *Plant and Cell Physiology*.

[37] Cannon SB, Zhu H, Baumgarten AM (2002). Diversity, distribution, and ancient taxonomic relationships within the TIR and non-TIR NBS-LRR resistance gene subfamilies. *Journal of Molecular Evolution*.

[38] Yang S, Feng Z, Zhang X (2006). Genome-wide investigation on the genetic variations of rice disease resistance genes. *Plant Molecular Biology*.

[39] Houb EB (2001). The arms race is ancient history in Arabidopsis, the wildflower. *Nature Reviews Genetics*.

[40] Głowacki S, Macioszek VK, Kononowicz AK (2011). R proteins as fundamentals of plant innate immunity. *Cellular and Molecular Biology Letters*.

[41] Takken FL, Albrecht M, Tameling WI (2006). Resistance proteins: molecular switches of plant defence. *Current Opinion in Plant Biology*.

[42] Muge Sayar-Turet SD, Braun HJ, Hede A, Dreisigacker S, MacCormack R, Boyd LA (2011). Genetic variation within and between winter wheat genotypes from Turkey, Kazakhstan, and Europe as determined by nucleotide-binding-site profiling. *Genome*.

[43] Kajava AV (1998). Structural diversity of leucine-rich repeat proteins. *Journal of Molecular Biology*.

[44] Bella J, Hindle KL, McEwan PA, Lovell SC (2008). The leucine-rich repeat structure. *Cellular and Molecular Life Sciences*.

[45] Stange C, Matus JT, Domínguez C, Perez-Acle T, Arce-Johnson P (2008). The N-homologue LRR domain adopts a folding which explains the TMV-Cg-induced HR-like response in sensitive tobacco plants. *Journal of Molecular Graphics and Modelling*.

[46] Kobe B, Kajava AV (2000). When protein folding is simplified to protein coiling: the continuum of solenoid protein structures. *Trends in Biochemical Sciences*.

[47] Ratnaparkhe MB, Wang X, Li J (2011). Comparative analysis of peanut NBS-LRR gene clusters suggests evolutionary innovation among duplicated domains and erosion of gene microsynteny. *New Phytologist*.

[48] Monosi B, Wisser RJ, Pennill L, Hulbert SH (2004). Full-genome analysis of resistance gene homologues in rice. *Theoretical and Applied Genetics*.

[49] Yu D, Chen C, Chen Z (2001). Evidence for an important role of WRKY DNA binding proteins in the regulation of NPR1 gene expression. *Plant Cell*.

[50] Li J, Brader G, Palva ET (2004). The WRKY70 transcription factor: a node of convergence for jasmonate-mediated and salicylate-mediated signals in plant defense. *Plant Cell*.

