# Supplementary material for: Genome Wide Analysis of Nucleotide-Binding Site Disease Resistance Genes in Brachypodium distachyon
Source: Comp Funct Genomics. 2012 May 28;2012:418208. doi: 10.1155/2012/418208 (PMC3368180; doi:10.1155/2012/418208)

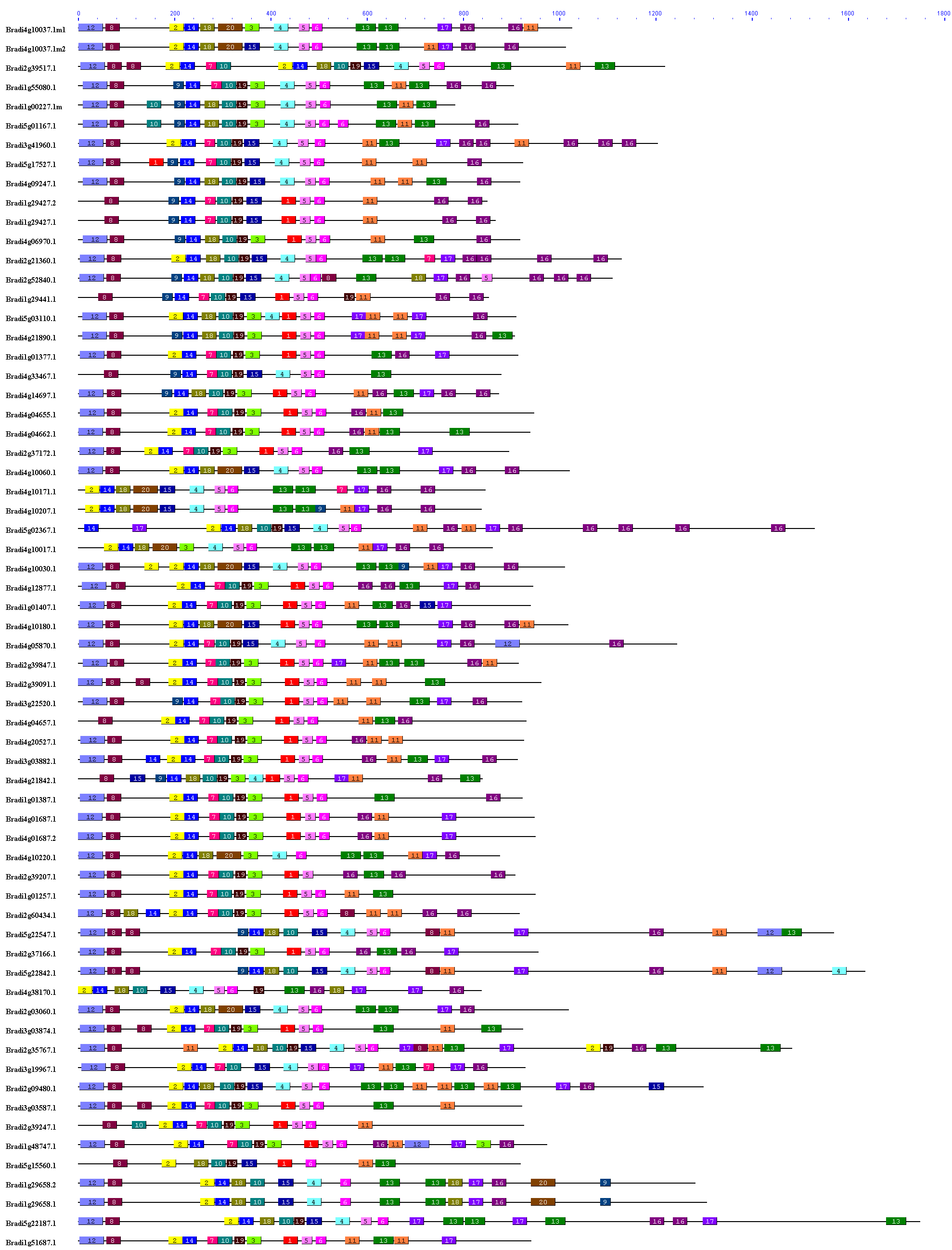

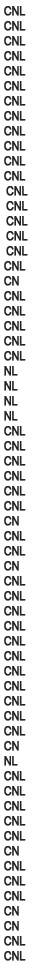
Supplemental file 3: Twenty putative motifs identified in the NBS family by MEME software. Different motifs are indicated by different colors. Types and names of the predicted 126 regular NBS-LRR genes are shown on the left side of the figure and the motif sizes are indicated at the top of the figure.


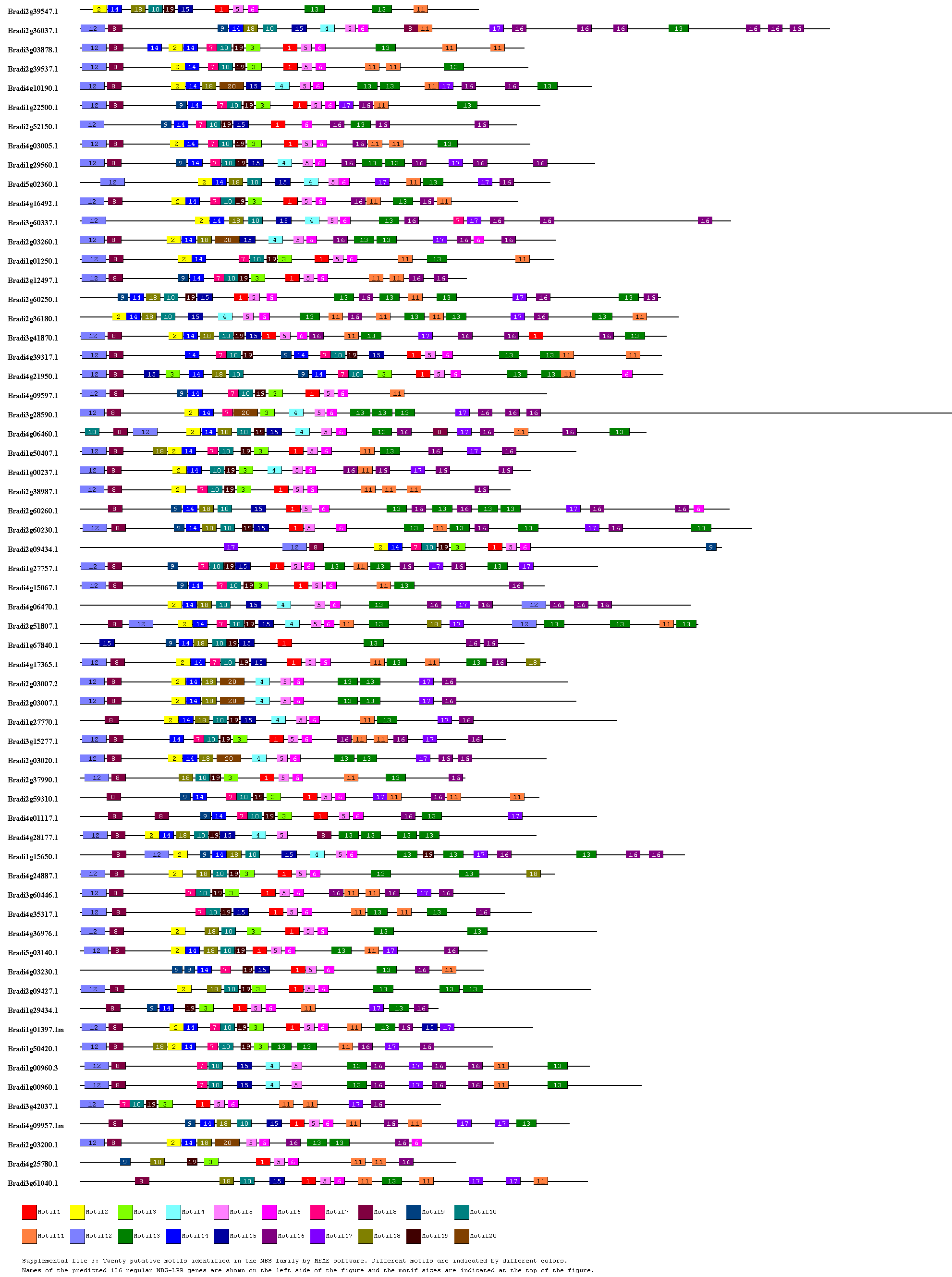

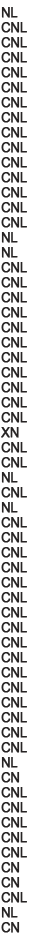

Supplement: Supplementary file 4 [file 418208.f4.doc]
